# Perspectives on Design Approaches for HIV Prevention Efficacy Trials

**DOI:** 10.1089/aid.2022.0150

**Published:** 2024-05-24

**Authors:** Holly J. Prudden, Roger Tatoud, Holly Janes, Stephaun Wallace, Veronica Miller, Linda-Gail Bekker, Deborah Donnell

**Affiliations:** ^1^ HIV Programmes and Advocacy, International AIDS Society, Geneva, Switzerland.; ^2^ Vaccine and Infectious Disease Division, Fred Hutch Cancer Center, Seattle, Washington, USA.; ^3^ Department of Global Health, University of Washington, Seattle, Washington, USA.; ^4^ Forum for Collaborative Research, UC Berkeley School of Public Health, Berkeley, California, USA.; ^5^ Desmond Tutu HIV Centre, Cape Town, South Africa.

**Keywords:** HIV prevention, clinical trial design, community engagement

## Abstract

The challenge of designing future HIV prevention efficacy trials in a rapidly evolving HIV prevention landscape was explored through a series of virtual stakeholder's engagement meetings convened online between October 2020 and April 2021. A broad array of stakeholders from the HIV prevention research community reviewed current trial designs and lessons learned, explored issues specific to unique product classes, and concluded with specialist-focused examinations of statistical design concepts and the importance of community engagement in research. The aim was to reflect on current approaches and evaluate new trial design approaches for evaluating efficacy of a candidate prevention strategy in the context of an active-controlled trial, which does not include a placebo arm. In this report, we provide a summary of the discussion points that included gaps in understanding and logical next steps in the prevention research pathway. The technical challenges involved in the statistical design approaches are described in a companion article.

## Introduction

HIV prevention is evolving rapidly with the rollout of new tools such as pre-exposure prophylaxis (PrEP) and treatment as prevention making a significant contribution toward controlling the HIV epidemic.^1,2^ These advances also change the highest priority questions in HIV prevention and as a result, impact the design and conduct of the next generation of prevention trials of candidate novel PrEP agents or delivery platforms, monoclonal antibodies (mAbs), and vaccines.^3^

Between October 2020 and April 2021, the Global HIV Vaccine Enterprise at the International AIDS Society (IAS), in collaboration with the HIV Vaccine Trials Network (HVTN), the HIV Prevention Trials Network (HPTN), and the Forum for Collaborative Research (UC Berkeley School of Public Health, USA) convened a series of virtual stakeholder's engagement meetings on Design Approaches for Current and Future HIV Prevention Efficacy Trials.

The event followed from a 2018 symposium in Seattle, Washington, sponsored by the HVTN, HPTN, Microbicide Trials Network (MTN), National Institute of Allergy and Infectious Diseases (NIAID/NIH), and Bill and Melinda Gates Foundation (BMGF),^4^ and was composed of three sessions, each consisting of live panel discussions supported by prerecorded presentations providing relevant background and ideas and introductions to new concepts (Table 1). All the materials for the symposium, including prerecorded talks and live recorded panel discussions, are freely available online from the HIV Global Vaccine Enterprise website, through the IAS (https://www.iasociety.org/ias-programme/global-hiv-vaccine-enterprise/virtual-workshop-series).

**Table 1:** : Overview of Event Series (Sessions 1–3): Individual Event Titles, Presenters, and Details on Accompanying Supporting Presentations

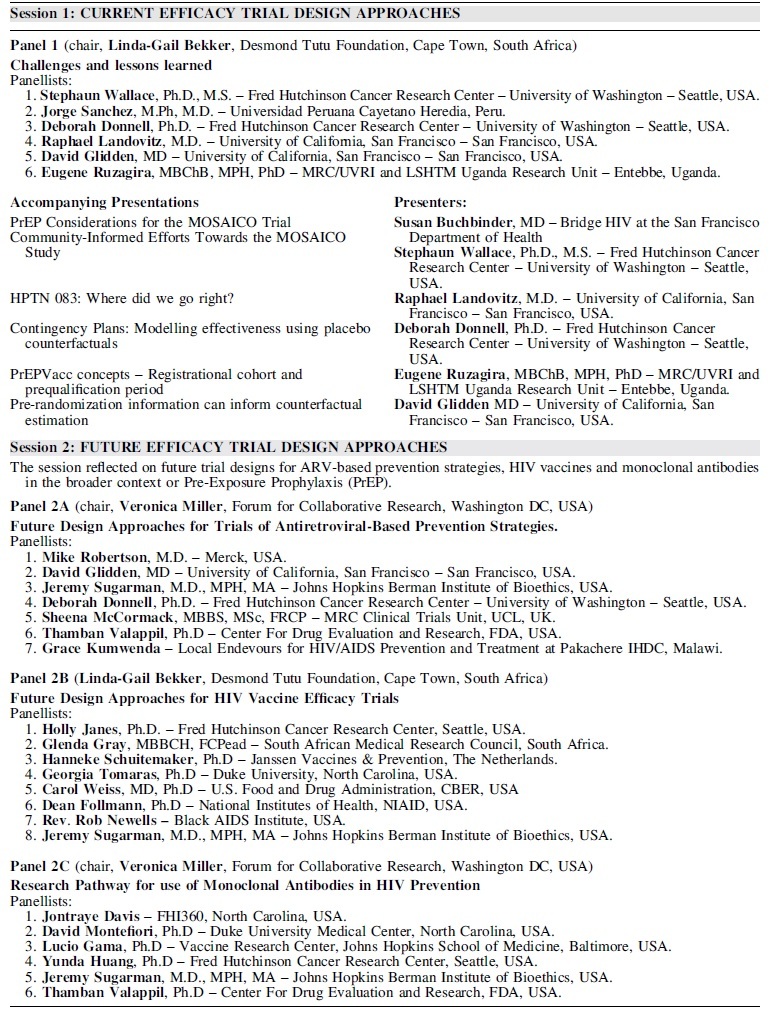 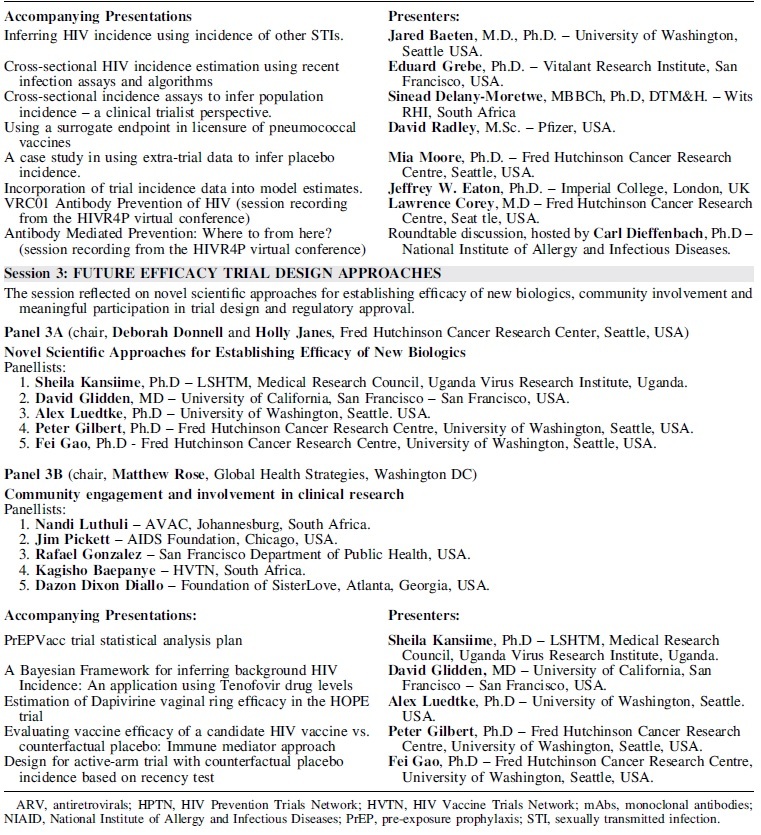

Participants reflected on recent advances and challenges within the HIV prevention field and on novel scientific approaches for establishing efficacy of new biologics in a future where all trial participants may have access to effective HIV prevention.

The focus was on future active-controlled HIV prevention trials, in which participants are randomized to an experimental intervention or active control. This is likely to be the norm for trials of interventions in classes with proven efficacy, such as antiretrovirals, and is on the horizon for trials of other interventions, such as mAbs and vaccines.

Two major challenges for active-controlled trials are ensuring adequate statistical power and interpreting the evidence of efficacy from an active-controlled trial in which there is low HIV incidence in both experimental and active-control.^3^

The presentation and panel discussions explored options for optimizing statistical power of active-controlled trials, augmenting the trials with external data for interpreting the evidence of efficacy of the experimental intervention, and alternatives to active-controlled trials.

## Current Clinical Trials: Challenges and Lessons Learned

Participants initially reflected on seminal past and current trial designs (Table 2) discussing their strengths and challenges. Efficacy trials to date have included a placebo control group or a registration cohort. Although the window of opportunity for placebo control trials may be closing because of the need to ensure access to the package of HIV prevention methods recommended by the World Health Organization (WHO),^5^ these studies can provide useful information for future trial design.

**Table 2.** Ongoing HIV Vaccine Efficacy Trials, as of July 2023

Study	Phase	Population	Regimen	Trials sites	Design	ClinicalTrials.gov identifier
HPX2008/HVTN705 IMBOKODO^a^	2b	2,600 women aged 18–35 years	Active immunization using Ad26.Mos4.HIV + Clade C gp140 vaccine for prime boost. Clade C gp140 only for boosters. Four injections over a 12-month period.	26 sites in 5 countries Africa	Placebo-controlled, randomized, double blind.	NCT03060629
HPX3002/HVTN706 MOSAICO^a^	3	3,800 HIV-negative men and transgender people who have sex with men, aged 18–60 years	Active immunization using Ad26.Mos4.HIV + Clade C and Mosaic gp140 vaccine, for prime and booster doses. Four injections over a 12-month period.	EU, Africa, South America	Placebo-controlled, randomized, double-blind—preferentially recruiting volunteers who do not want to take PrEP	NCT03964415
PrEPVAcc	2b	Up to 1,668 adults (18–40 years)	Two experimental vaccine regimens: DNA-HIV-PT123 + monomeric AIDSVAX B/E and DNA-HIV-PT123 + CN54gp140 + MVA CMDR (weeks 0, 4, 24, 48) and DNA/CN54gp140 (weeks 0, 4)+MVA/CN54gp140. Four injections over 48 weeks.	Four African countries	Three-arm, two-stage HIV prophylactic vaccine trial, with concurrent open-label randomization to daily TAF/FTC or TDF/FTC PrEP (weeks 0–26). Placebo-controlled, randomized, double-blind.	NCT04066881

^a^ Vaccinations stopped due to futility.

TAF, Tenofovir alafenamide; TDF/FTC, Tenofovir disoproxil fumarate/emtricitabine.

### HVTN 705 and HVTN 706

The Imbokodo (HVTN 705/HPX2008) phase 2b and MOSAICO (HVTN706/HPX3002) phase 3 studies evaluated the efficacy of a heterologous prime-boost vaccine regimen utilizing the Adenovirus-26 platform and aluminum phosphate-adjuvanted Clade C gp140 and Mosaic gp140 envelop protein for the prevention of HIV infection in HIV-negative women, and HIV-negative men and transgender people who have sex with men, respectively.

Enrolment into MOSAICO (HVTN 706) was conditional upon the nonuse of daily PrEP at the start of the study. Potential participants already or planning to use PrEP were excluded from enrolment. However, once enrolled and after receiving their first vaccination, should participants change their mind they were allowed to take PrEP according to the site PrEP plan and would continue to receive further vaccinations. This study design approach allows researchers to maintain a “true” placebo group, thereby mitigating the effect of a highly effective prevention method, that would otherwise mask the impact of any vaccine should it be effective—while ensuring this is achieved within ethical guidelines.

Work to ensure community involvement in the study was key to its feasibility, with effective community and stakeholder engagement ensuring acceptability of the approach. No commonalities were identified among those who choose not to take PrEP in this trial, but it was noted that an important factor in the decision to participate in the study was how countries approached the long-term provision of PrEP, and particularly confidence in PrEP being available outside of the study. Strong community support and participation in MOSAICO have enabled a trial design that includes a true placebo group. However, with current trials enrolling ∼5,000 participants, there is now a strong motivation to explore other ways of achieving sound evidence for efficacy.

### The PrEPVacc trial

The PrEPVacc trial is a multi-arm multi-stage adaptive prophylactic HIV vaccine trial with a second randomization to compare Tenofovir alafenamide (F/TAF) with Tenofovir disoproxil fumarate/emtricitabine (TDF/FTC) PrEP. Two experimental combination vaccine regimens are compared with a placebo control with an open-label randomization comparing daily TAF/FTC with daily TDF/FTC as PrEP. The study differs in its approach from that of HVTN 705 and HVTN 706, in that all groups are provided with daily oral PrEP (TDF/FTC or TAF/FTC) over weeks 0–26 during the vaccination period, to protect against intercurrent infection. Rather than enrolling a placebo group, the study instead enrolled participant in a *registrational cohort* to obtain an estimate of baseline incidence in the population (Box 1), which acts as a comparator.

**Box 1.** Explanation of the Use of a “Registrational Cohort”

**What is a registrational cohort and what is its purpose?** A registrational cohort is a pretrial stage during which participants are enrolled in a clinical study, but where no drugs are evaluated. Participants are tested regularly for HIV infection, which provides information on the baseline HIV incidence in the community where the study will take place. **What are the advantages of a registration cohort?** First, to identify, recruit, and follow-up a cohort of volunteers at risk of HIV who may be willing to participate in the trial. Second, to provide an estimate of the incidence of HIV in the community. This is crucial as it allows researchers to calculate the potential effect of PrEP in the trial. It is one method of calculating, what is known as a “counterfactual placebo.” Third, it allows researchers to understand people's knowledge, perceptions, uptake of, and adherence to PrEP (where it is available). Finally, to engage community in defining and refining key messages about HIV vaccines and PrEP, as well as developing tools to convey these messages.

Information from https://www.prepvacc.org/registration-cohort

PrEP, pre-exposure prophylaxis.

Using a registrational cohort comes with the advantage of being able to engage the community before the trial starts. This is particularly important when the concept of the trial itself has a more complex design. In addition, the cohort represents a “same time, same place, and same population group,” which act as a counterfactual for the trial. This approach also allows people who do not enter the trial to continue to benefit from being part of the research. Additionally, the registrational cohort does provide an opportunity to assess whether suitable populations are enrolled in the study and if incidence levels are adequate to power the trial.

### Key considerations for the use of registrational cohorts

The reliability of the registrational cohort and its representativeness were identified as a potential issue. The risk of acquiring HIV within the cohort may decrease during a run-in period (as higher risk individuals acquire HIV), although it is not clear if this would be the case.

Extra study cost and approval from national regulatory agencies that data obtained from a registrational cohort can be used for regulatory purpose and may also affect funders' willingness to support a study if the latter is not acceptable to the regulator. As a standalone, it may be difficult to secure funding for registrational cohorts that are not tied to a trial.

During the discussions, panelists also acknowledged that registrational cohorts are subjected to the potential bias of regional differences, which would require running multiple registration cohorts in various locations. In this case, the covariates that are different mean that it may be difficult to justify the constancy assumptions.

If registrational cohorts do become standard practice, a key question from a community perspective will be what to expect regarding the provision of HIV prevention tools while participants “wait” for the trial to begin. This will likely differ depending on where the study is conducted, with an additional consideration being how to collect more information during this period that could inform future designs and future products, for example, what would be acceptable if daily PrEP in its current format is not available?

## Future Clinical Trial Designs

Several future statistical design approaches were discussed at the workshop regarding how to establish efficacy of an experimental prevention agent, in future trials, when you can no longer employ a placebo arm, and existing forms of prevention which will be required as part of trials are highly effective. These statistical design approaches are fully described with examples: assumptions, strengths, and weaknesses; and additional future statistical research needs to ensure unbiased estimates of efficacy in a separate article.^6^ In this report, we list the approaches, which either provide an HIV incidence rate to be used as “counterfactual placebo” (i.e., a credible estimated placebo that can be compared with the intervention being tested) or estimate efficacy using a correlate of protection.

(1) Those eligible for a planned active-controlled trial are followed longitudinally in a registrational cohort until the start of the trial, and HIV incidence is measured.
(2) Recency assays are used during trial enrolment to estimate background HIV incidence among participants screened for an active-controlled trial.
(3) HIV incidence is assessed from historical or contemporary external trials that include a placebo arm.
(4) Biomarkers of HIV exposure risk (e.g., sexually transmitted infections such as rectal gonorrhea) are used to infer HIV incidence.
(5) Efficacy of the active control as used in the active-control trial is estimated based on the relationship with a biomarker of adherence (established in prior placebo-controlled trial.
(6) Efficacy of an experimental vaccine or monoclonal antibody is estimated using an immunological marker of efficacy, established in prior placebo-controlled trials to mediate efficacy of the intervention.

## Community Perspective on Current and Future Trials

As research advances and effective HIV prevention becomes more available, the design of efficacy trials becomes more complex and placebo-controlled studies are becoming unjustifiable. During the online panel discussions, community stakeholder representatives reflected on current and emerging perceptions relating to the conduct of trials and how scientific research will be conducted in the future. Effectively and meaningfully engaging with all stakeholders in the design and conduct of biomedical HIV prevention trials has long been recognized as central to successful research.^3^ Stakeholders are an integral part of research, and framing the conversation about trial design from an end user perspective should be central to both the design of prevention products and to the research itself, to ensure successful research and uptake.

Throughout the panel discussions, four key themes emerged from the conversation with community representatives: knowledge, representation, collaboration, and engagement.

### Supporting learning and building research literacy in communities

Motivations to participate in clinical research are diverse, and studies have described the positive and negative social impact associated with participation in clinical research.^4^ The concept of “the lived experience,” as a means of describing all facets of research participant involvement, was presented as an important way to understand community participation in clinical studies. Community members felt that this component whether qualitative or quantitative in nature should be considered a necessary feature of the science in future trials. A key focus for this would be in supporting the implementation phase where the need for and acceptability of new products could be measured.

Community representatives expressed the need to be considered as experts in the scientific process, beyond supporting recruitment activities. Supporting and developing research literacy among communities where research takes place ensures community is engaged in the research and motivated to participate. The provision of educational material both at the start of trials and throughout the trials has been shown to be effective at generating a sense of excitement and improved participation and retention in the study. New and more complex trial designs, such as in PrEPVacc and its registrational cohort, and MOSAICO and its “layered” approach, emphasized the need to build and improve research literacy within participating communities. Communities should play a leading role, and the participation of a broad range of stakeholders, beyond trial participants, is required with everyone learning together. Panelists noted the need for a shared language and shared understanding of these new concepts as crucial to the success of clinical research.

### Community participation in research

Participation in clinical research is an integral part of participants' everyday life and must be understood in this broader context. A better knowledge and a greater understanding of the motivation and of the impact of participating in clinical research can help identifying and mitigating potential negative impacts of participating in research while encouraging contribution and improving the chance of success to develop a product that will be acceptable to the end users. From the onset, there is a strong desire within communities participating in clinical research to better understand the product development pipeline and the potential decisions and trade-offs leading to different clinical trial modalities. While acceptability of products was seen as important, desirability of products was seen as most critical. Ensuring this is achieved before trials began will provide information that will benefit the scientific process.

### Community engagement in the wider context

Community engagement needs to happen at every step of the research in future trials, from product and trial design to the conduct of a trial, and results dissemination. Ensuring communities understand and play an active role in informing the wider prevention context increases the willingness to contribute to clinical research programs. Different levels of engagement were discussed: the local context where the study takes place; the regional or national context, where decisions are made regarding who benefits; and the global context, where discussions on wider and universal access to products and product acceptability and desirability occur. Further ensuring a wider range of demographics is included in research to ensure confidence that an effective and an approved product would be adopted within different communities was also emphasized. Meaningful collaborations that go beyond representation will be central to enabling clinical research to provide more options and choices.

Examples within the San Francisco Community Engagement Team illustrated how the establishment of the team within the community in every space has cultivated trust and promoted participation.^7,8^ The careful focus on key populations and their needs, as illustrated by these programs, builds stronger relationships and trust, leading to better outcomes both within and beyond the research.

## Summary and Next Steps

Building on the 2018 HIV Prevention Efficacy Trial Designs of the Future symposium, the 2021 series of stakeholder's engagement events allowed for further explorations of the growing challenges of conducting HIV prevention efficacy trials in the context of effective HIV prevention. In this report, we described past and current trial designs, highlighted new challenges including reaching field-wide consensus and acceptability of new approaches, and emphasized the importance of meaningful community involvement and engagement in future trials. A separate article will discuss the ethical and statistical challenges faced by future trials. Future engagement events should continue to assess and reflect on rapid changes in the field, as well as continuing to work closely with communities toward building much needed research literacy.
